# The Epidemiological Impact of Community-Based Skin Camps on Leprosy Control in East Hararghe Zone, Ethiopia: a Modelling Study

**DOI:** 10.1007/s44197-025-00370-5

**Published:** 2025-03-04

**Authors:** Thomas Hambridge, David J. Blok, Ephrem Mamo, Jan Hendrik Richardus, Sake J. de Vlas

**Affiliations:** 1 Department of Public Health, Erasmus MC, University Medical Center Rotterdam, Rotterdam, The Netherlands; 2 Aklilu Lemma Institute of Pathobiology (ALIPB), Addis Ababa University, Addis Ababa, Ethiopia; 3 Armauer Hansen Research Institute (AHRI), Addis Ababa, Ethiopia

**Keywords:** Leprosy, Epidemiology, Case detection delay, Skin camps, Mathematical modelling

## Abstract

**Background:**

Leprosy is a chronic infectious disease that remains a public health challenge in many low- and middle-income countries. The mainstay of leprosy control has been early detection and treatment through active case finding. In this study, we aimed to predict the epidemiological impact of community-based skin camps to shorten the period of leprosy case detection delay in a population.

**Methods:**

We used the individual-based model SIMCOLEP to predict the epidemiological impact of two successive community-based skin camps with 50%, 70% and 90% target population coverage conducted five years apart (in 2024 and 2029). The model was calibrated to the leprosy situation in East Hararghe zone, Ethiopia (2008–2023).

**Results:**

There was a short-term rise in the new case detection rate due to a backlog of cases being discovered, but no difference in the long run compared to the baseline situation (i.e., no intervention). However, all strategies substantially decreased the prevalence of undiagnosed symptomatic cases in the population. Skin camps with 50% coverage resulted in 21.8% (95% CI: 20.1–23.5%) fewer cases per million in 2035, while increasing the coverage to 90% led to a reduction of 33.0% (95% CI: 31.5–34.4%) in 2035. This impact was sustained for the skin camps with 90% coverage, with a 30.9% reduction compared to baseline in 2040.

**Conclusion:**

Our findings suggest that shortening the period of leprosy case detection delay through community-based skin camps could substantially reduce the prevalence of symptomatic cases in high endemic regions, leading to improved disease control.

**Supplementary Information:**

The online version contains supplementary material available at 10.1007/s44197-025-00370-5.

## Introduction

Leprosy, also known as Hansen’s disease, is a chronic infectious disease caused by *Mycobacterium leprae* [1]. Although significant progress has been made over the past few decades, the disease remains a public health problem in several countries [2]. *M. leprae* is slow growing and has a very long incubation period, ranging from 1 to more than 20 years [3]. As a result, one of the main challenges in leprosy control is early detection and prompt treatment of cases, which can help prevent transmission and reduce disability among affected individuals [4, 5]. Leprosy classification at diagnosis is based on clinical signs according to World Health Organization (WHO) criteria. The disease is classified as paucibacillary (PB) when five or less skin patches with loss of sensation are present, and multibacillary (MB) when there are six or more skin patches with loss of sensation and/or an affected nerve [6].

The introduction of multidrug therapy (MDT) in the 1980s led to a substantial decline in prevalence and the WHO declaring leprosy eliminated as a public health problem globally in the year 2000. However, the decline was due to the shortened treatment duration and the removal of treated patients from national registries, rather than to a substantial decrease in incidence [7, 8]. Early diagnosis and treatment can not only mitigate the progression of the disease in the infected individual, but also plays a key role in interrupting the transmission cycle.

Case detection delay (CDD) is defined as the time from first signs of leprosy until diagnosis [9]. This period of CDD in leprosy cases remains a major obstacle to effective control, and there is a need to explore interventions that can shorten it [10, 11]. In 2020, the post exposure prophylaxis for leprosy (PEP4LEP) trial was initiated in Ethiopia, Mozambique and Tanzania to compare two integrated skin screening interventions combined with single-dose rifampicin as post-exposure prophylaxis (SDR-PEP). The main screening intervention is community-based, using skin camps to screen approximately contacts of leprosy cases, while the other intervention is health centre-based, inviting household contacts to be screened in a local health centre. The community-based skin camps are organised when a patient is diagnosed with leprosy and approximately 100 people from the same community living in the surrounding area are invited for skin screening for leprosy and other skin-NTDs. These skin camps are designed to bring specialised care closer to the community through close collaboration with community leaders and local organisations, with health workers assisted by a dermatologist, thus expanding healthcare access. Moreover, the integrated skin screening approach promoted by the WHO is believed to enhance the effectiveness of the intervention by expanding coverage of individuals at higher risk of developing leprosy, as it targets those living in rural and lower socioeconomic areas that are disproportionately affected by skin-NTDs [12, 13]. In PEP4LEP, case detection delay will be used as the main outcome measure of effectiveness [14]. A baseline survey was carried out in each country as part of the study to collect estimates of CDD in patients diagnosed between 2020 and 2021 through interviews with a structured questionnaire within six months of diagnosis. In Mozambique and Tanzania, the mean CDD reported from the PEP4LEP baseline survey was 26.6 months and 28.1 months, respectively [15, 16]. In Ethiopia, the survey was conducted in three endemic districts located in East Hararghe zone in the Oromia Region of Ethiopia: Girawa, Jarso, and Midega. The mean detection delay in these districts was 22.4 months, based on data from 50 recently diagnosed patients [17]. Another recent study of 97 leprosy patients diagnosed in the same region reported a mean delay of 22.5 months [18].

In this modelling study, we focused on Ethiopia, which is listed as one of the WHO’s 23 global priority countries and reported 2395 new leprosy cases in 2023 [2]. The new case detection rate (NCDR) of leprosy in East Hararghe zone has been steadily declining over the past 15 years. However, the zone remains highly endemic with 312 new cases (75.7 per million population) reported in 2023 [19]. To predict the epidemiological impact of shortening leprosy CDD through active case finding in East Hararghe zone, Ethiopia, we used the individual-based model SIMCOLEP. This model simulates the transmission and control of leprosy (including novel interventions) in a population structured by households. SIMCOLEP has been used in several recent studies to model various leprosy control scenarios, including new diagnostic tests, contact tracing, and SDR-PEP [20–23]. Here, we calibrated the model to historical leprosy case data with the aim to predict the epidemiological impact of two community-based skin camps with 50%, 70% and 90% coverage of the target population conducted five years apart.

## Methods

### Model Description

The stochastic individual-based transmission model SIMCOLEP was used to simulate the spread of *M. leprae* in East Hararghe zone, Ethiopia [24, 25]. The model simulates the life histories of individuals, defined by birth, death, marriage, household formation, movement between households, as well as infection, disease development and control measures. Transmission of *M. leprae* can occur when an infectious individual comes in contact with a susceptible individual. Two transmission processes are modelled separately: transmission in the general population and within households, with the latter reflecting an increased risk of becoming infected. Transmission is determined by the product of the contact rate, both in the general population (c_pop_) and within households (c_hh_), and the infectivity function [24]. The contact rate represents the frequency that an infectious individual gets into contact with others in the population or household. Two leprosy subtypes are distinguished in the model: PB leprosy, which is self-healing and not considered infectious, and MB leprosy, representing chronic infection and considered infectious. The infectivity function increases linearly from 0 to 1 throughout the asymptomatic phase and remains constant at 1 for the symptomatic phase. When an individual is infected, they develop either PB or MB leprosy, which is determined based on the observed MB proportion in the population. As part of the baseline conditions, the model also incorporates existing control measures in the population, namely treatment of all new leprosy cases with MDT, and the protective effect of BCG vaccination in the population prior to infection.

### Model Quantification

Pre-set model parameters were based on independent sources, including demographic and epidemiological data. A complete overview of the model parameters and demographic data used for quantification can be found in Supplementary Information, Table A. We quantified the population using area-specific or national demographic data, including projected population size and growth rate, age distribution, age specific fertility rates, survival rates, fraction married, and distribution of household size [26–28]. The MB/PB ratio from East Haraghe zone leprosy case data was 75/25 [19]. The model and natural history of leprosy used follows previous studies, with a full description available in Fischer et al*.* 2010 and Blok et al*.* 2015 [25, 29]. In the model, we assume that only 20% of individuals are susceptible to leprosy and that 80% will not develop leprosy [29]. Every infected individual who is diagnosed is administered MDT and no longer transmits to the rest of the population following their treatment (100% treatment completion). The protective effect of BCG vaccination in infants prior to infection was set to 60% [30], and coverage estimates were obtained from WHO/UNICEF Estimates of National Immunization Coverage (ranging from 5% in 1981 to 91% in 2020) [31]. The within household effective contact rate c_hh_ was fixed to the value used in previous studies (i.e., 0.98 per year), with the assumption that household contact rates are similar across different settings [29, 32].

A series of calibration rounds were performed to assess model fit using combinations of fixed pre-set parameters described above and free parameters that varied within a given range. Firstly, free model parameters were calibrated for demographic processes against household size distribution, including fraction of individuals creating own household and household size to move to. Secondly, two free parameters for disease processes were estimated against leprosy NCDR per million in East Hararghe zone from 2008 to 2023: population effective contact rate c_pop_ and mean detection delay. The latter refers to the duration of time between first symptom and diagnosis as a leprosy case in the model population and was assumed to follow a log-normal distribution in line with a recent study that found variation in detection delays are best described by this type of probability distribution [33]. The distribution has two parameters: the mean and variance. During the calibration process, we varied the mean from 1 to 6 years for 1998 onwards, before the start of the fitted NCDR time period. The variance was fixed to 1.5 years. Historical mean detection delays were based on previously published model calibrations [29, 32]. We calibrated the model using NCDR up to 2023, following the short-term epidemiological impact of COVID-19 (specifically on leprosy incidence) in many parts of the world due to interruption of leprosy control activities [34–36]. We conducted a grid search for the two disease parameters. We compared the simulated NCDR to the observed NCDR using a log-likelihood function assuming a Poisson distribution. For each parameter combination, we based our outcomes (i.e., simulated NCDR) on the mean of 20 stochastic runs. The 100 best fitting parameter combinations for each calibration round were selected. The outcomes of these parameter combinations were then used to calculate the mean, 2.5th percentile and 97.5th percentile to generate confidence intervals (reflecting uncertainty of parameter estimates). All predictions were based on these 100 best fitting parameter combinations.

### Scenarios

Three scenarios of two successive community-based skin camp interventions in 2024 and 2029 were implemented using three different coverage levels. In PEP4LEP skin camps, recently diagnosed leprosy cases are registered as index patients and integrated skin screening is offered to contacts in their community [14]. Given the large number of new leprosy cases found during screening of contacts at these skin camps, with more than half presenting with an existing skin condition, we assumed that these interventions are highly targeted towards those most at risk of developing leprosy [37]. To date, there were 12, 10 and 16 new leprosy cases diagnosed through PEP4LEP skin camps in Jarso, Midega and Girawa in 2020, representing around 40–50% of the total number of new cases detected in each area. Therefore, we modelled skin camp scenarios with a basic coverage level of 50%, but also a more ambitious 70% and 90%, to investigate the potential full impact of shortening CDD through active case finding. As a comparator, we modelled the impact of the baseline situation in which no interventions outside of MDT and BCG vaccination in infants were introduced. Model outcomes include NCDR per million and the prevalence of undiagnosed cases. In addition, we assessed the impact of alternative parametrisations of pre-intervention detection delays (i.e., mean and variance) on these outcomes in a sensitivity analysis. Predictions were made until 2040, with trend lines representing an average of each 100 best fitting parameter combination. Analyses performed in this study adhered to the criteria specified by the Policy-Relevant Items for Reporting Models in Epidemiology of Neglected Tropical Diseases (PRIME-NTD) (Supplementary Information, Table C) [38].

## Results

The simulated leprosy NCDR per million was plotted against the observed NCDR data in East Hararghe zone between 2008 and 2023. Modelled historical trends of the 100 best fitting parameter combinations and with 95% CIs can be found in Supplementary Information, Fig A. The model simulations provided a good fit to the historical observed data and were therefore used for predictions of interventions to shorten the period of CDD in the population after 2023. A gradual declining trend in NCDR is observed in East Hararghe zone over this period in the baseline situation, typical of many leprosy endemic areas globally and driven mainly by MDT treatment of passively detected cases.

**Fig. 1:**
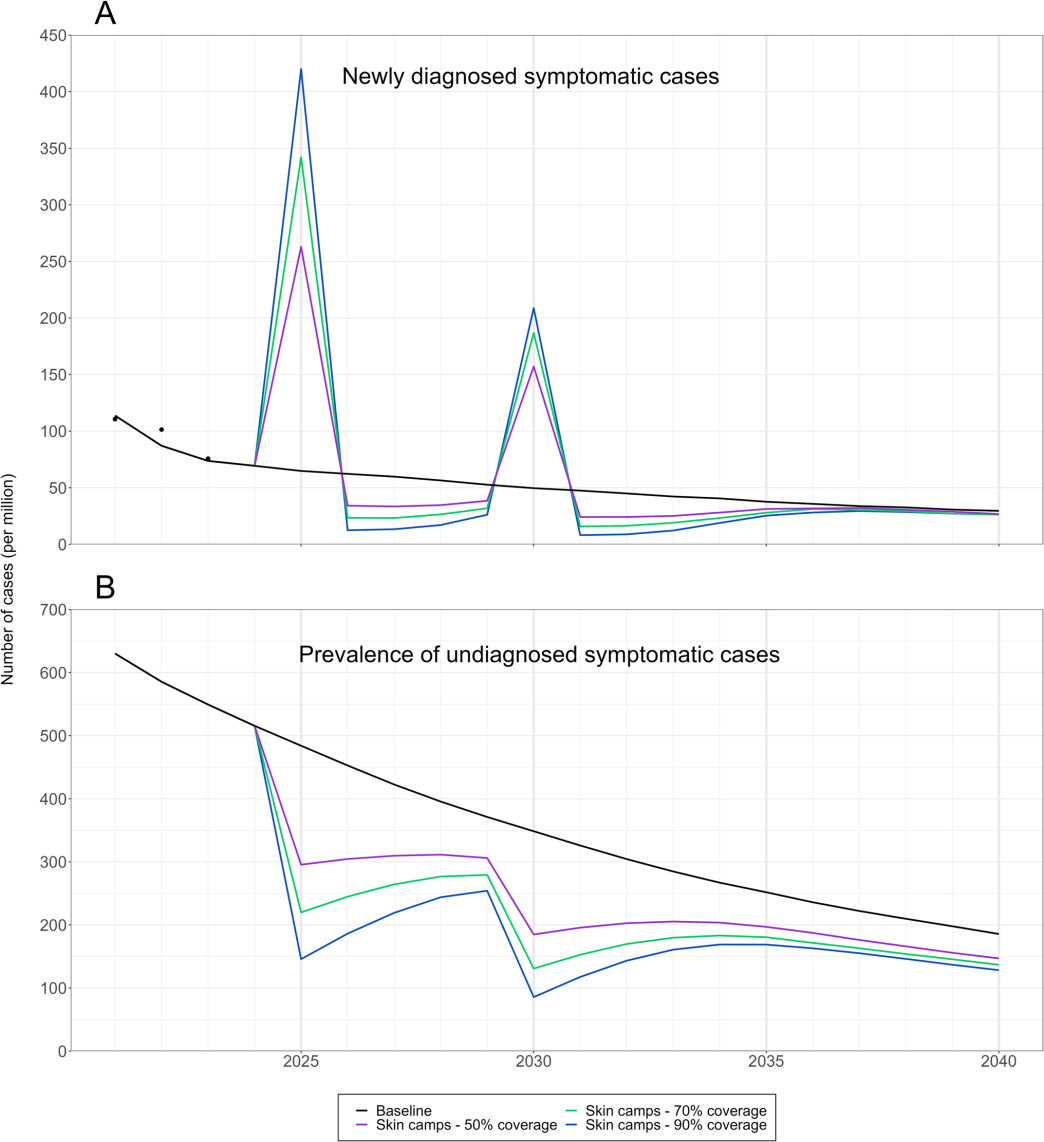
Predicted epidemiological impact of shortening leprosy case detection delay. **A** Model scenario projections of newly diagnosed symptomatic cases per million up to 2040. The black dots indicate observed historical NCDR data in East Hararghe zone, Ethiopia. **B** Model scenario projections of the prevalence of undiagnosed symptomatic cases per million up to 2040. Three scenarios are shown for successive skin camps with 50% (purple), 70% (green) and 90% (blue) target population coverage, with the black line representing the baseline situation. Results are an average of the outcomes of the 100 best fitting parameter combinations

When implementing the active case finding scenarios through community-based skin camps, trends in the number of newly diagnosed symptomatic cases first showed an increase, demonstrating the diagnosis (and treatment) of backlog cases in the population (Fig. 1). A large increase was observed directly following implementation of two skin camps, peaking at 420 (95% CI: 415–425) and 209 (95% CI: 205–212) cases per million respectively in 2025 and 2030 when coverage of the target population was 90%. However, there was no significant difference in NCDR at 2040 compared to the baseline situation for any of the interventions.

The predicted prevalence of undiagnosed cases was 549 (95% CI: 542–557) per million in 2023, approximately 7–8 times higher than the NCDR. Two successive skin camps with 50% coverage of the target population resulted in 197 (95% CI: 194–200) undiagnosed symptomatic cases per million in 2035, i.e., 21.8% (95% CI: 20.1%–23.5%) fewer than the baseline. With 90% coverage, the reduction was significantly greater at 169 (95% CI: 166–172) in 2035, i.e., 33.0% (95% CI: 31.5%–34.4%) fewer undiagnosed cases. The epidemiological impact of skin camps with a high coverage of 90% was sustained: 128 (95% CI: 126–130) undiagnosed symptomatic cases per million compared to 186 (95% CI: 183–189) in the baseline situation in 2040 (30.9% reduction). All results were robust to alternative parameterisations for pre-intervention detection delays (sensitivity analysis in Supplementary Information, Table B).

## Discussion

Case detection delay (CDD) in leprosy is an important epidemiological indicator for reducing transmission and preventing disability. Although the new case detection rate of leprosy in East Hararghe zone, Ethiopia has been gradually declining over the past 15 years, the zone remains highly endemic. It is therefore imperative to implement strategies to shorten the period of CDD to reduce the number of leprosy cases over time. Our study shows that interventions that diagnose and treat leprosy cases with MDT earlier, such as community-based skin camps with high target population coverage, can have a substantial epidemiological impact.

The predictions from our model indicate that if CDD is shortened though active case finding in skin camps, first a peak in NCDR will be observed due to a backlog of cases being discovered, followed by a decline to similar levels as the baseline scenario. Nevertheless, the prevalence of undiagnosed cases has substantially decreased compared to the baseline scenario. Skin camps with 50% coverage of the target population (in line with the leprosy detection rate observed in PEP4LEP to date) resulted in 22% fewer cases per million in 2035. Increasing the coverage to 90%, representing an ideal situation with highly effective contact screening, led to a reduction of 33% in 2035. This illustrates the importance of reducing detection delays, as it can reduce the number of undiagnosed cases (who are left untreated) and as a result reduce transmission. However, it is important to note that achieving a 90% (or even 50%) coverage scenario may be highly challenging if not unfeasible in remote areas endemic for other NTDs.

The scenarios presented, while theoretical, align with the screening interventions implemented in the PEP4LEP study. The skin camp coverage levels of 50%, 70% and 90% were derived from preliminary study findings and are considered ambitious but also attainable, as evidenced by the number of new leprosy cases detected so far in PEP4LEP skin camps carried out in the Ethiopian study districts, which represent around 40–50% of the total number of new cases detected in each area [37]. Because skin camps require a strong partnership with community leaders and local organisations, they tend to be well received [39]. Moreover, the comprehensive approach to skin screening helps maintain confidentiality for individuals with leprosy, thus diminishing the associated stigma [40].

Our findings have important implications for not only introducing community-based integrated skin screening, but also other interventions for enhanced case finding. These include field-based diagnostics, mobile health (mHealth) tools to support peripheral health workers, and contact tracing for recently diagnosed leprosy patients, which can also shorten detection delay in the population [41–43]. Screening for multiple skin diseases (including leprosy and other skin-NTDs) is recommended by the WHO to enhance effectiveness and efficiency by minimising costs and expanding coverage [12, 13, 44]. To investigate this further, the cost-effectiveness of the integrated screening approach in the context of skin camps is a secondary outcome measure of the PEP4LEP study [14]. On a practical level, as leprosy incidence declines, maintaining these shortened delays will likely become more challenging due to health workers and the general population having less experience recognising the signs and symptoms of leprosy [5]. It is therefore also imperative to continue developing sustainable programmes in the community such as leprosy education and awareness campaigns, involvement of traditional healers, and enhanced training of healthcare workers for diagnosis of skin-NTDs [13, 45, 46].

The accuracy of our model predictions depends on the validity of the NCDR data from East Hararghe zone that were used to fit the model, as well as the uncertainty around some of the assumptions in SIMCOLEP regarding leprosy transmission. While we were able to quantify the model using national data from the Ethiopian Ministry of Health, new leprosy case and population data were only available in East Hararghe from 2008 and the true prevalence of undiagnosed leprosy cases in any population is unknown [19]. While the same is true for the period of CDD, we introduced a log-normal distribution for this parameter in the SIMCOLEP model as it has been shown that variation of leprosy case detection delay data is best described by this type of probability distribution [33]. Movement is also an important consideration for an individual-based transmission model such as SIMCOLEP and migration was not accounted for in our study. Many people in Oromia region and specifically East Hararghe are pastoralists, relying on livestock herding and moving across different areas to make use of resources, which may have consequences on transmission that are difficult to quantify [47–49]. The model also does not account for environmental and socioeconomic factors known to be associated with leprosy. For instance, leprosy mainly affects marginalised populations and stigma can lead to isolation and delays in diagnosis for persons affected. Taking measures to mitigate this, such as integrating community education and awareness of leprosy and NTDs, is important to maintain participation and high coverage levels for these types of screening interventions. We used individual coverage levels for skin camp scenarios that, as described earlier, were based on the assumption that mostly high risk groups are targeted, but in reality these interventions take place in areas with high numbers of reported cases and not randomly throughout the population. While we believe the predicted impact of these interventions is relevant to other high endemic areas and countries, especially in East Africa, context-specific differences such as healthcare infrastructure and demographics must be considered in future modelling studies to enhance applicability. Furthermore, the feasibility of achieving such high coverage levels in real-world settings and the cost-effectiveness of skin camps, which are highly resource intensive particularly in remote areas, must be further investigated as secondary outcomes. Our study did not account for any additional leprosy control activities that may be ongoing in East Hararghe zone, such as chemoprophylaxis for contacts of leprosy patients in PEP4LEP study areas.

## Conclusion

Leprosy case detection delay is an important indicator for interruption of transmission and preventing disability. Our model demonstrated that community-based skin camps to shorten detection delay can substantially reduce the number of leprosy cases in the long term. Our model scenarios, which ranged from modest to ambitious in target population coverage are realistic in terms of operational capabilities and resource availability in high-endemic regions. We recommend implementing skin camps with integrated NTD screening in high endemic populations as an effective disease control strategy.

## Supplementary Information

Below is the link to the electronic supplementary material.

Supplementary file1 (DOCX 325 KB)

## Data Availability

The modelling data and relevant analytical code underlying this article will be shared on reasonable request to the corresponding author.
