# Supplementary material for: The Epidemiological Impact of Community-Based Skin Camps on Leprosy Control in East Hararghe Zone, Ethiopia: a Modelling Study
Source: J Epidemiol Glob Health. 2025 Mar 4;15(1):34. doi: 10.1007/s44197-025-00370-5 (PMC11880465; doi:10.1007/s44197-025-00370-5)
Supplement: Supplementary file 1 — Supplementary file1 (DOCX 325 KB) [file 44197_2025_370_MOESM1_ESM.docx]

**Supplementary Material**

**Contents**

[Table A. Overview of parameters used in the model. 2](#_Toc160551779)

[Fig A. Model fitting of new leprosy case detection rates to the observed data in East Hararghe zone, Ethiopia (individual parameter combinations). 3](#_Toc160551780)

[Table B. Sensitivity analysis for detection delay (DD) parameters: mean and variance. 4](#_Toc160551781)

[Table C. Policy-Relevant Items for Reporting Models in Epidemiology of Neglected Tropical Diseases (PRIME-NTD) [7]. 5](#_Toc160551782)

[References 6](#_Toc160551783)

**Table A. Overview of parameters used in the model.**

| **Parameter** | **Value** | **Source** |
| --- | --- | --- |
| **Natural history of infection** | | |
| **Proportion susceptible** | 20% (random mechanism) | Fischer et al. 2010 [1] |
| **MB proportion** | 75% | East Hararghe zone - National Leprosy Data 2023 [2] |
| **Contact rate** | | |
| **General population (c_pop_)** | 0.0588 (95% CI: 0.0575 – 0.0597) | Calibrated |
| **Within household (c_hh_)** | 0.98 | Fischer et al. 2010 [1] |
| **Control** | | |
| **BCG protective effect** | 60% | Rodrigues, et al. 2007 [3] |
| **BCG coverage range** | 5% (1981) **-** 91% (2020) | WHO/UNICEF Estimates of National Immunization Coverage 2020 [4] |
| **Case detection delay (years)** | | |
|  | **1970 onwards:** Mean = 50, var = 33 (log-normally distributed) | Assumption |
|  | **1985 onwards:** Mean = 12, var = 8 (log-normally distributed) | Fischer et al. 2010 [1] |
|  | **1995 onwards:** Mean = 7, var = 5 (log-normally distributed) | Fischer et al. 2010 [1] |
|  | **1998 onwards:** Mean = 5, var = 2 (log-normally distributed) | Calibrated |
|  | **2020 onwards:** Mean = 5, var = 1.5 (log-normally distributed) | Calibrated |
| **Household movement** | | |
| **Distribution of household size** | Household size rural Ethiopia 2016 (mean size = 4.9 members) | Ethiopia Demographic and Health Survey 2016 [5] |
| **Household size to move to** | Triangular distribution (start = 0, end = 7, max = 2) | Calibrated |
| **Time until splitting of a married household from parental household** | Mean = 12 (exponentially distributed) | Assumption |
| **Demographics** | | |
| **Projected population growth rate** | Exponential annual growth rate = 2.34% (2000 to 2050) | UN World Population Prospects 2022 [6] |
| **Average fertility rates** | **1950:** 7.32  **1990:** 7.22  **2015:** 4.51 | UN World Population Prospects 2022 [6] |


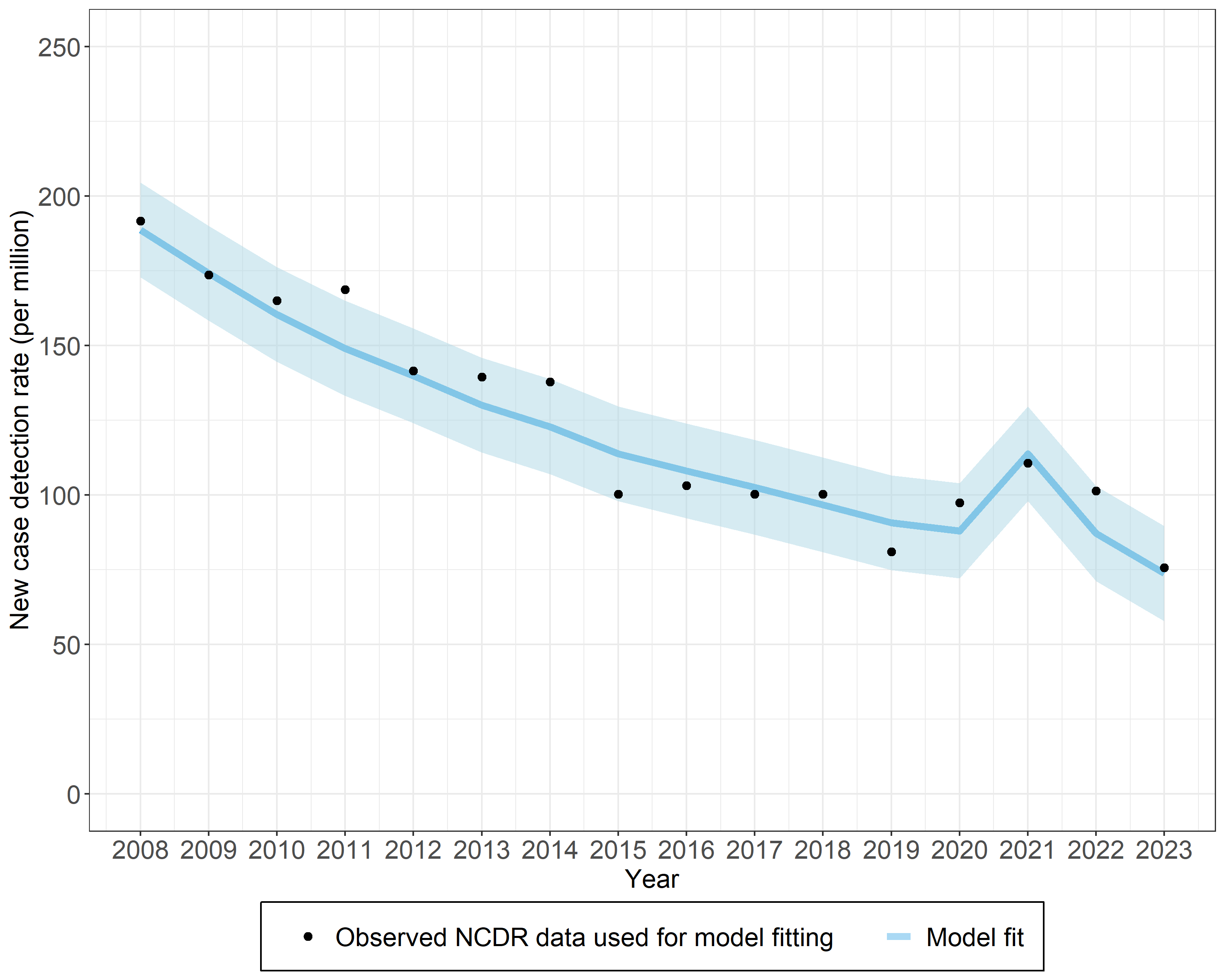


**Fig A. Model fitting of new leprosy case detection rates to the observed data in East Hararghe zone, Ethiopia.** Modelled historical trends of 100 best fitting parameter combinations are shown with leprosy new case detection rates in East Hararghe zone, Ethiopia from 2008 - 2023. Observed yearly data are represented by black dots. The solid blue line represents the mean model estimate and the shaded area represents the 95% prediction interval reflecting variation in population contact rates and detection delay.

**Table B. Sensitivity analysis for detection delay (DD) parameters: mean and variance.** Comparison of model outputs (newly diagnosed cases and undiagnosed prevalence per million) to calibrated detection delay distribution parameter values used in the main results (mean = 5 years / variance = 1.5 years, shaded below in grey) pre-intervention. The changes shown below represent the difference (total and %) in number of cases in 2040 compared to baseline. All other parameter values (mb proportion, c_pop_, c_hh_) in the model were fixed according to the overview in Table A.

| **Skin camps - 90% coverage** | | | | | | |  |
| --- | --- | --- | --- | --- | --- | --- | --- |
| **DD parameters** | **Newly diagnosed cases**  **(per million) in 2040** | | | **Undiagnosed prevalence**  **(per million) in 2040** | | |  |
|  | **N  (baseline)** | **N  (intervention)** | **Change**  **(%)** | **N  (baseline)** | **N  (intervention)** | **Change**  **(%)** |  |
| Main results DD mean = 5 years DD var = 1.5 years | 30 | 26 | -4 (-11.1) | 186 | 128 | -58 (-30.9) |  |
|  |  |  |  |  |  |  |  |
|  |  |  |  |  |  |  |  |
| Lower mean (4 years) | 29 | 25 | -4 (-13.7) | 162 | 102 | -60 (-36.7) |  |
|  |  |  |  |  |  |  |  |
|  |  |  |  |  |  |  |  |
| Higher mean (6 years) | 30 | 26 | -5 (-15.2) | 222 | 160 | -62 (-27.8) |  |
|  |  |  |  |  |  |  |  |
|  |  |  |  |  |  |  |  |
| Lower variance (1 year) | 30 | 26 | -4 (-13.4) | 196 | 126 | -70 (-35.6) |  |
|  |  |  |  |  |  |  |  |
|  |  |  |  |  |  |  |  |
| Higher variance (2 years) | 30 | 24 | -7 (-21.6) | 201 | 142 | -59 (-29.3) |  |
|  |  |  |  |  |  |  |  |
|  |  |  |  |  |  |  |  |

**Table C. Policy-Relevant Items for Reporting Models in Epidemiology of Neglected Tropical Diseases (PRIME-NTD) [7].**

| **Principle** | **What has been done to  satisfy the principle?** | **Where in the manuscript is this described?** |
| --- | --- | --- |
| **Stakeholder engagement** | Methodology and findings communicated to relevant stakeholders, including PEP4LEP consortium and international leprosy NGOs. | - |
| **Complete model documentation** | The model and calibration process is described, with a full overview of parameters used in the model and reference to SIMCOLEP studies [1] for model assumptions based on previous exploratory analyses. | Methods section and references, Supplementary Material: Table A. |
| **Complete description of data used** | An overview of model parameter values and demographic data used to quantify the model are provided, as well as the data source. Values and/or the specific time/periods used from each data source are also presented. | Supplementary Material: Table A. |
| **Communicating uncertainty** | The uncertainty of parameter estimates is reflected in the 95% prediction intervals. A sensitivity analysis of detection delay parameter combinations was also performed to demonstrate the effect of incremental changes. | Results section, Supplementary Material: Table B. |
| **Testable model outcomes** | The model outcomes can be tested by implementing future interventions in East Hararghe zone, Ethiopia and observing the impact on NCDR, including the future epidemiological trends observed in the PEP4LEP study. | Discussion section. |

**References**

1. Fischer E, De Vlas SJ, Meima A, Habbema D, Richardus JH. Different mechanisms for heterogeneity in leprosy susceptibility can explain disease clustering within households. PLoS One. 2010; 5(11), e14061.

2. Ethiopian Ministry of Health. East Hararghe zone - National Leprosy Data 2023.

3. Rodrigues LC, Kerr-Pontes LR, Frietas MV, Barreto ML. Long lasting BCG protection against leprosy. Vaccine. 2007; 25(39-40), 6842-6844.

4. World Health Organization. WHO/UNICEF estimates of national immunization coverage. 2020.

5. Central Statistical Agency/CSA/Ethiopia, & ICF. Ethiopia Demographic and Health Survey. 2016.

6. United Nations. World population prospects. 2022.

7. Behrend, MR, Basáñez, MG, Hamley, JI, Porco, TC, Stolk, WA, Walker, M, et al. Modelling for policy: the five principles of the Neglected Tropical Diseases Modelling Consortium. PLoS Negl Trop Dis. 2020; 14(4), e0008033.
